# Transcriptional analysis identifies potential biomarkers and molecular regulators in pneumonia and COPD exacerbation

**DOI:** 10.1038/s41598-019-57108-0

**Published:** 2020-01-14

**Authors:** Wilhelm Bertrams, Kathrin Griss, Maria Han, Kerstin Seidel, Andreas Klemmer, Alexandra Sittka-Stark, Stefan Hippenstiel, Norbert Suttorp, Florian Finkernagel, Jochen Wilhelm, Timm Greulich, Claus F. Vogelmeier, Julio Vera, Bernd Schmeck

**Affiliations:** 1 Institute for Lung Research, Universities of Giessen and Marburg Lung Center, German Center for Lung Research (DZL), Marburg, Germany; 2 Department of Internal Medicine/Infectious Diseases and Respiratory Medicine, Charité – University Medicine Berlin, Berlin, Germany; 3 Pulmonary and Critical Care Medicine, University Medical Center Giessen and Marburg, German Center for Lung Research (DZL), Marburg, Germany; 4 Institute of Molecular Biology and Tumor Research (IMT), Genomics Core Facility, Philipps-University of Marburg, Marburg, Germany; 5 Justus-Liebig-University, Universities Giessen & Marburg Lung Center, German Center for Lung Research (DZL), Giessen, Germany; 6 Laboratory of Systems Tumor Immunology, Department of Dermatology, Universitätsklinikum Erlangen and Friedrich-Alexander-Universität Erlangen-Nürnberg, Erlangen, Germany; 7 Center for Synthetic Microbiology (SYNMIKRO), Philipps-University of Marburg, Marburg, Germany; 8 German Center for Infection Research (DZIF), partner site Giessen-Marburg-Langen, Marburg, Germany

**Keywords:** Microarrays, Molecular medicine

## Abstract

Lower respiratory infections, such as community-acquired pneumonia (CAP), and chronic obstructive pulmonary disease (COPD) rank among the most frequent causes of death worldwide. Improved diagnostics and profound pathophysiological insights are urgent clinical needs. In our cohort, we analysed transcriptional networks of peripheral blood mononuclear cells (PBMCs) to identify central regulators and potential biomarkers. We investigated the mRNA- and miRNA-transcriptome of PBMCs of healthy subjects and patients suffering from CAP or AECOPD by microarray and Taqman Low Density Array. Genes that correlated with PBMC composition were eliminated, and remaining differentially expressed genes were grouped into modules. One selected module (120 genes) was particularly suitable to discriminate AECOPD and CAP and most notably contained a subset of five biologically relevant mRNAs that differentiated between CAP and AECOPD with an AUC of 86.1%. Likewise, we identified several microRNAs, *e.g*. miR-545-3p and miR-519c-3p, which separated AECOPD and CAP. We furthermore retrieved an integrated network of differentially regulated mRNAs and microRNAs and identified HNF4A, MCC and MUC1 as central network regulators or most important discriminatory markers. In summary, transcriptional analysis retrieved potential biomarkers and central molecular features of CAP and AECOPD.

## Introduction

Community acquired pneumonia (CAP) is clinically defined by a sudden onset of severe illness that is accompanied by signs of lower respiratory tract infection, fever, cough and dyspnoea^1^. When left untreated, severe secondary effects such as organ damage and occurrence of bacteria in the blood (bacteremia) can ensue. While subject to variance due to region, season and population characteristics, the incidence of CAP is estimated to lie between 1.5 and 14 cases per 1,000 persons per year, with children under 5 years of age and the elderly of more than 65 years being most strongly affected^2^. Immunocompromised persons also bear a higher risk of CAP contraction.

The leading underlying cause of CAP is infection with the gram-positive bacterium *Streptococcus pneumoniae*, accounting for 30–35% of CAP cases worldwide^3^. The initial colonization of the nasopharynx and the upper respiratory tract often remains asymptomatic. Aspiration into the alveoli can cause severe respiratory or systemic disease, depending on the host immune status and the pneumococcal serotype^1^.

As CAP is a multifaceted disease with a host of potential causative agents, the robust identification of microRNAs that are functionally involved in pneumonia depends on the pathogen. In severe Influenza A Virus (H1N1) related pneumonia, *e.g*., miR-150 was significantly up-regulated in serum, whereas the expression of miR-210, miR-126 and miR-222 was decreased, albeit the small sample size precludes definitive conclusions^4^.

Chronic obstructive pulmonary disease (COPD) is a poorly reversible condition that is characterized by airflow limitation and subsequent decrease of lung function^5^. It is linked to an abnormal inflammatory response of the respiratory tract and the lung to noxious particles and gases. Disease manifestations are cough, sputum production and dyspnoea. Genetic susceptibility (a1-antitrypsin deficiency), severe respiratory infections, neutrophilic childhood asthma or prolonged exposition to chemicals and dust pose additional risk factors for COPD.

The pathology of COPD is defined by destruction of lung parenchyma (resulting in emphysema) and obstruction of the small airways (resulting in obstructive bronchiolitis). Comorbidities of COPD include cardiovascular diseases, osteoporosis and lung cancer. Acute exacerbations (AE) are episodes of aggravated COPD symptoms and often co-occur with respiratory infection. Furthermore, exacerbations increase the patient’s propensity for new exacerbations^6,7^. Exacerbations might require hospitalization, and they increase the COPD mortality rate^8^. This deleterious disease development mandates the search for biomarkers that can identify AECOPD.

The regulation of microRNAs in the lung during COPD or in patients at risk to develop COPD has been described in several studies. Alveolar macrophages^9^, induced sputum^10^, lung tissue^11^ and lung fibroblasts^12^ have been analysed. In most cases, these studies found decreased levels of microRNAs. In alveolar macrophages from smokers vs. non-smokers, e.g., the number of down-regulated microRNAs in the smoker cohort was larger than the number of up-regulated microRNAs^9^. Likewise, 27 of 34 differentially expressed microRNAs in induced sputum between smokers and never-smokers were decreased in current smokers^10^. In serum, microRNA let-7c was down-regulated in COPD patients, and miR-34c was decreased in smokers with or without COPD^13^, which matched results from sputum^10^. miR-199a-5p has been shown to be down-regulated in regulatory T cells of COPD patients^14^. Among the microRNA candidates that show an upregulation in COPD lung tissue are miR-15b and miR-144 in lung tissue^11^. The same study revealed that in total lung tissue of smokers with and without COPD, 57 microRNAs were up-regulated and 13 microRNAs were down-regulated in patients with COPD compared to healthy smokers, thus contrasting with studies that suggest a comprehensive miRNA downregulation in COPD.

Therefore, we comprehensively analysed both protein coding mRNA and regulatory miRNAs in clinically accessible leukocytes in order to identify markers that not only inform about pathophysiology but may also be suitable as diagnostic biomarkers. We collected PBMCs from healthy donors and patients with either CAP or acute exacerbation of COPD and found very distinct miRNA and mRNA expression pattern, also when compared to healthy controls. Computational analysed retrieved differentially regulated RNA circuits with HNF4A, MCC, MUC1, miR-545-3p, and miR-519c-3p as important parts.

## Methods

### Patient samples

Patients suffering from community-acquired pneumonia or acutely exacerbated COPD were recruited immediately after hospitalization. In addition, healthy subjects were recruited (Tables 1 and S1). Inclusion criteria of community-acquired pneumonia patients included pulmonary infiltrates on chest x-ray and clinical presentation. Inclusion criteria of COPD patients with an acute exacerbation (AECOPD) were an acute respiratory worsening requiring a hospitalization in pre-diagnosed COPD, but without pulmonary infiltrates on chest x-ray or highly elevated CRP-levels (Table S1). Only one CAP patient presented with a diagnosis of COPD. No other CAP patient showed clinical signs or a medical history of COPD. Immunosuppressed, pregnant and HIV-positive patients were excluded from the study. The BioInflame study was approved by the ethics committee of the Charité - Universitätsmedizin Berlin (EA2/030/09) and the University Medical Center Marburg (55/17). All blood donors were at least 18 years of age and provided written informed consent for use of their blood samples for scientific purposes. PBMCs were isolated by Pancoll gradient centrifugation of one collected Vacutainer EDTA-tube (6 ml whole blood). All methods were performed in accordance with the relevant guidelines and regulations.

**Table 1:** Basic characteristics of the BioInflame study cohort.

	Healthy	CAP	AECOPD
**MicroArray Cohort**			
mean age [years ± SD]	43.8 ± 12.61	75 ± 7.26	62.67 ± 8.84
gender m/f (%)	1/4 (20/80)	4/2 (66.6/33.3)	3/3 (50/50)
pack years ± SD	0	30 ± 19.75	72.5 ± 57.86
n	5	6	6
**TLDA Cohort**			
mean age [years ± SD]	44.9 ± 11.3	65.6 ± 13.6	62.7 ± 9.9
gender m/f (%)	3/4 (42.9/57.1)	4/3 (57.1/42.9)	3/4 (42.9/57.1)
pack years ± SD	0	14.29 ± 14.25	67.86 ± 54.50
n	7	7	7

### Microarray and Taqman Low Density Array

PBMC samples from healthy donors, AECOPD patients and CAP patients were obtained via gradient centrifugation of 6 ml whole blood. Cells were lysed in TriReagent (Invitrogen, Carlsbad USA), and RNA was extracted by Phenol/Chloroform precipitation. Gene expression was measured on an Agilent Sureprint mRNA array as described before^15^, using an Agilent SurePrint G3 Human Gene Expression 8 × 60 K v2 Microarray kit with 200 ng of RNA per sample. Background correction was performed with the normexp model using negative control spots and offset of 1. Spot intensities were quantile normalized between arrays. Array quality was assessed using spatial hybridization homogeneity, per-probe intra-array signal variation and per-probe median-to-mean ratios. Contrasts were fitted using limma. Genes that were significantly regulated (unadjusted p < 0.01) in either of the contrasts AECOPD vs. healthy, CAP vs. healthy or AECOPD vs. CAP were selected (Table S2). Genes whose expression changes could be attributed to changes in cellular composition of the PBMC fractions between donors (Table S3) were removed from the list of significantly regulated genes. To this end, linear modeling was applied using statsmodels^16^ ordinary least squares regression (OLS-function). Gene expression was standardized to run from 0 to 1, and modelled as a linear combination of disease states (boolean: AECOPD/CAP) and cellular composition (real valued: neutrophils/eosonophils/monocytes/lymphocytes in %) plus an error term. The data are deposited under the accession number GSE94916. Expression data from the independent reference dataset GSE6269^17^ was used as deposited in GEO. Data were obtained via the getGEO function from the GEOquery package^18^ in R. Contrasts were fitted using limma. Genes were considered to be differentially expressed when unadjusted p < 0.05.

Taqman Low Density Arrays (TLDAs) were applied to reveal disease-related-regulation of microRNAs as described before^19^. The data are deposited under the accession number GSE136390.

### Bioinformatics

The R programming software^20^ was used with the packages Weighted Gene Co-expression Network Analysis (WGCNA) v. 1.66^21^, Ensemble Feature Selection (EFS) v. 1.0.3^22^, gplots v. 3.0.1 and ggplot2 v. 3.1.0.9. WGCNA identifies expression modules in a given dataset by correlating gene expression data across samples^21^. Gene groups (modules) were established on the basis of high adjacency and subsequent gene-tree cutting with a minimum module size of 30 and maximum height of 1. A weighted average for each module (the module eigengene, which is the first principal component of the correlated genes in a given module) was used to compute a measure of module membership (kME) for each gene in the module. kME values represent the correlation between a gene’s expression and the module eigengene (ME). WGCNA was used here to find modules that allow discrimination between CAP and AECOPD. Biological connections of identified factors were investigated with Ingenuity Pathway Analysis^23^ and visualized with Cytoscape v. 3.7.1^24^. Hub genes from the identified discriminatory module (module I) were ranked by their contribution to disease separation using EFS.

Gene expression intensities of the independent dataset GSE6269^17^ were probed for enrichment of module I genes by Gene Set Enrichment Analysis (GSEA)^25^, available at http://software.broadinstitute.org/gsea/index.jsp.

## Results

### Differential gene expression in CAP and AECOPD as compared to healthy donors can be detected in PBMCs

**Figure 1:**
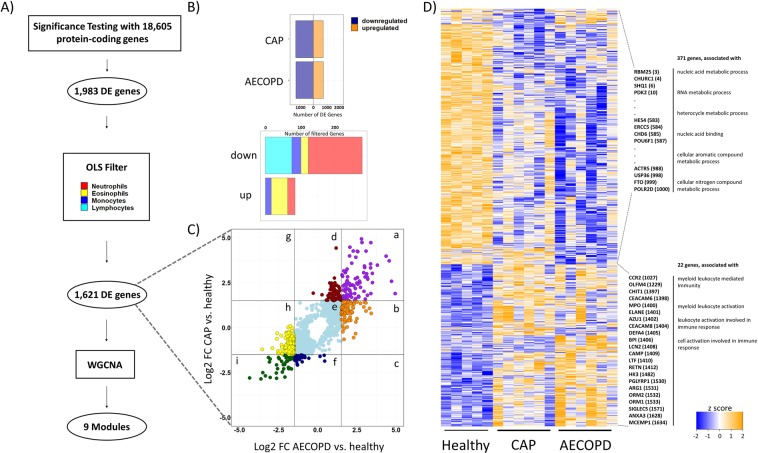
CAP and AECOPD have an impact on the PBMC transcriptome. Schematic diagram of the workflow (**A**). From a total number of 1,983 pairwise significantly differentially expressed (DE) genes between the coding transcriptomes of PBMCs from AECOPD or CAP patients or healthy controls, genes whose expression changes were attributable to changes in cell number were removed (OLS filter) (**B**). The remaining 1,621 genes that were significantly differentially expressed are shown by their log_2_ fold change vs. healthy control (**C**). All 1,621 are shown in a z-score representation. Enriched GO terms (−log_10_ pvalue > 5) and exemplary genes that drive this enrichment are indicated with their position (in brackets) in the heatmap. Gene ranking was achieved by hierarchical clustering (**D**).

We probed the PBMC transcriptome of healthy donors, AECOPD patients and CAP patients for potential biomarkers by microarray and Taqman Low Density Array. As illustrated (Fig. 1A), coding transcripts that were significantly regulated (unadjusted p < 0.01) in the contrasts CAP vs. healthy, AECOPD vs. healthy or CAP vs. AECOPD were identified (Table S2). Then, these genes were correlated to the PBMC composition of the individual donors to filter out gene expression changes that were attributable to a change of PBMC cellular composition (Fig. 1B), which removed 362 of 1,983 genes (Table S3). While these genes hold potential as biomarkers, they are not suited for functional network building, and they might be strongly influenced by composition of the drawn blood sample. The remaining coding transcripts (1,621, Table S4) tend to broadly follow similar regulation patterns in CAP and AECOPD when compared to healthy controls (Fig. 1C). All 1,621 DE genes are shown in a z-score representation and were probed for GO term enrichment. To this end, they were separated into genes that were up-regulated in disease or down-regulated in disease. The strongest enrichments (−log_10_ pvalue > 5) are shown including exemplary genes that were driving this enrichment (Fig. 1D). Detailed z-score heatmaps of the genes from the six populated outer regions of Fig. 1C, sorted by hierarchical clustering, are given in the supplement section “Appendix to Fig. 1”.

### Correlation analysis yields a subset of genes that can discriminate CAP from AECOPD and is also applicable to an independent PBMC transcriptomic dataset

**Figure 2:**
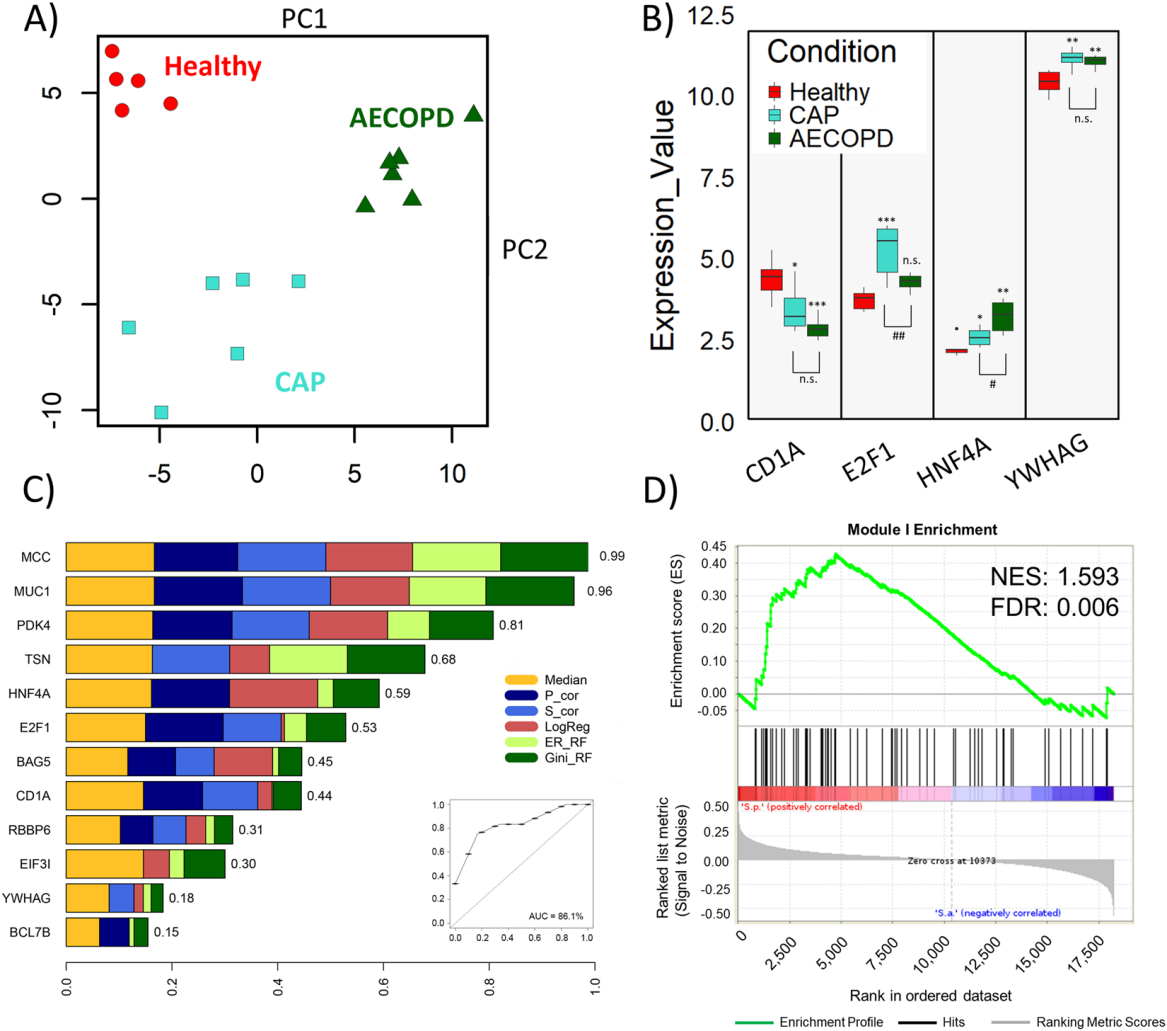
Module I is highly discriminatory for CAP/AECOPD. Module I genes effectively separate healthy donors (red) from CAP (turquoise) and AECOPD patients (green) in a PCA. Data were centred and scaled before analysis (**A**). Microarray expression values of module I hub genes, which were identified as such by Ingenuity Pathway Analysis, show differential expression as a function of disease status. The boxes define the first and third quartile, whiskers extent at most 1.5*IQR (interquartile range) from the hinge. The horizontal bar represents the median (**B**). Hub genes and connecting genes from module I were analysed by Ensemble Feature Selection (EFS) for their potential to differentiate between CAP and AECOPD. We used the default EFS algorithm, which ranks feature importance as an additive value comprised of median comparison (p values from Wilcoxon signed rank test), S_Cor (Spearman’s rank correlation test by fast correlation filter), P_Cor (Pearson’s product moment correlation test by fast correlation filter), LogReg (beta-Values of logistic regression), ER_RF (Error-rate-based variable importance measure embedded in random forest) and Gini_RF (Gini-index-based variable importance measure embedded in random forest). The plot shows the relative normalized importance for each individual method plus the combined performance of MCC, MUC1, PDK4, TSN and HNF4A in a ROC analysis (**C**). Genes from module I positively correlate with blood leukocyte DE (p < 0.05) mRNA from *Streptococcus Pneumoniae*-infected patients from an independent study (*GSE6269*) when compared to *Staphylococcus Aureus*-infected patients from the same study. NES = Normalized enrichment score. FDR = False Discovery Rate (**D**).

**Figure 3:**
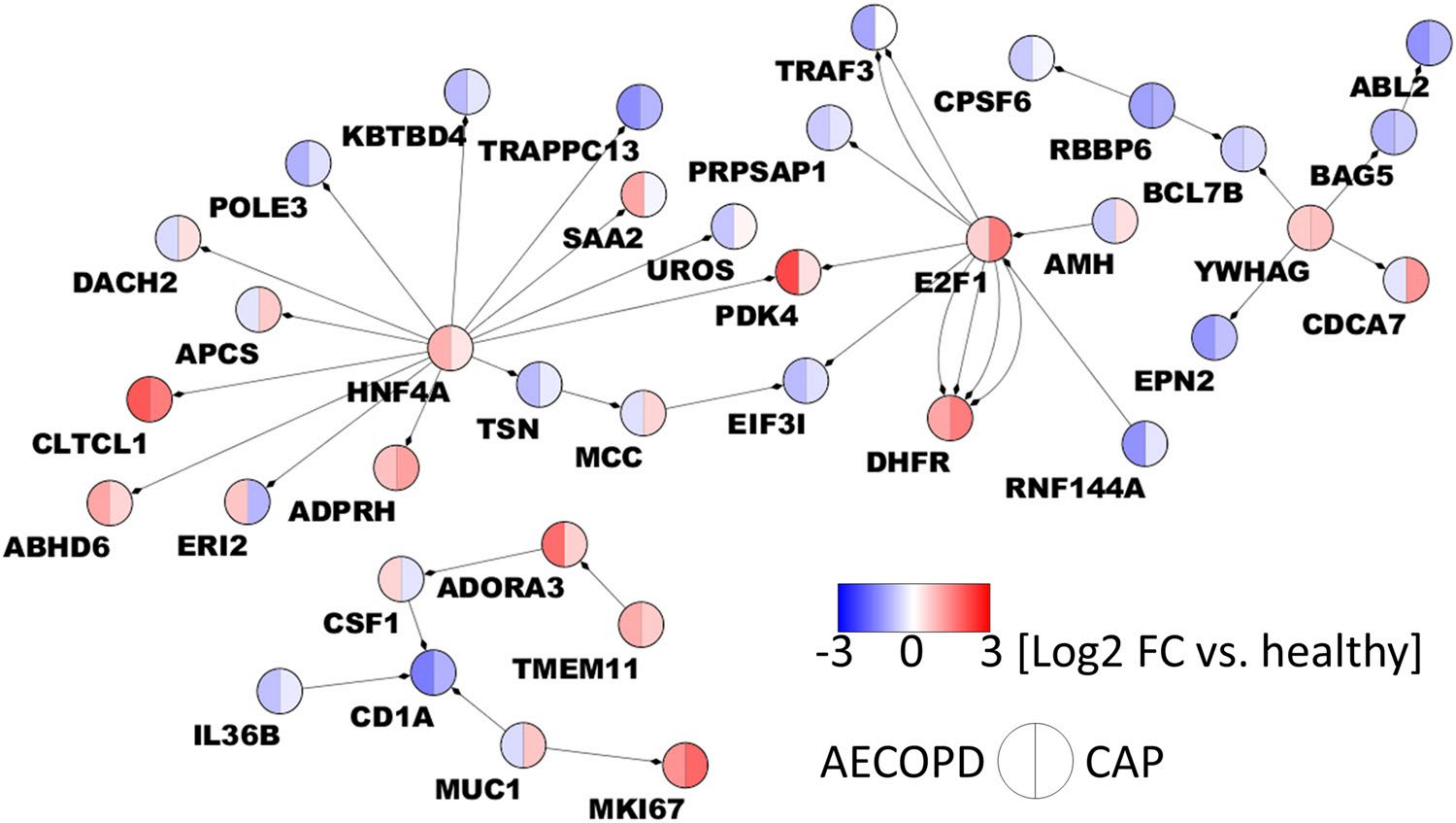
Central network constructed on the basis of genes included in module I. Ingenuity Pathway Analysis was used to integrate module I biologically. Parameters of interaction screening were set to only include experimentally validated candidates from the *human* and *uncategorized* species settings. Orphan nodes and isolated node pairs were deleted. Log_2_ fold expression changes vs. healthy control are shown for AECOPD (left semicircle) and CAP (right semicircle). The network was visualized with Cytoscape v. 3.7.1.

In order to test the disease-discriminatory potential of the complete gene set from Fig. 1, we applied PCA analysis which successfully clustered the healthy and disease phenotypes, but failed to properly resolve differences between the CAP and AECOPD samples (Fig. S1). Therefore, we applied supervised Weighted Gene Co-expression Network Analysis (WGCNA) to this subset of 1,621 significantly regulated coding genes. Parameters were iteratively set to yield the largest possible modules while still preserving a given gene set’s ability to separate AECOPD from CAP. We tested each module Eigengene for its correlation with disease phenotype (CAP or AECOPD). We finally identified nine modules, the bottom module IX serving as collector for genes that could not be assigned to any of the other modules (see file modules_expression_values.xlsx). Module I with 120 genes (Table S5) showed by far the most significant and clear discrimination between CAP and AECOPD (Fig. S1), and consequently discriminated these disease subtypes in PCA, maximizing the distances to nearest neighbours from different clusters (Fig. 2A and Table S6). In module I, correlation between the individual gene’s expression and the module Eigengene was robust for the module genes (R^2^ = 0.609, Fig. S2), highlighting the overall stability of gene expression within this module and its suitability for disease discrimination. Module hub genes (Fig. 2B) were identified by Ingenuity Pathway Analysis on the basis of their connectivity with other members from module I. In an additional step, all genes with at least two intra-module interacting partners were probed by Ensemble Feature Selection in order to find genes with high importance in separating CAP from AECOPD and biological relevance in the module. We thereby ranked the identified genes by their contribution to the model and identified five out of twelve genes that are the most important discriminators (EFS selected features: MCC, MUC1, PDK4, TSN and HNF4A) and that perform with an AUC of 86.1% in a ROC analysis (Fig. 2C). Furthermore, we tested module I on an independent dataset of blood leukocytes from patients with acute infections, Gene Expression Omnibus accession number GSE6269^17^. We used Gene Set Enrichment Analysis (GSEA)^25^ to probe for enrichment of module I genes in this dataset, and we found a marked positive enrichment of these genes when comparing *S. pneumoniae* with *S. aureus* infected patients (Fig. 2D), strengthening the applicability of module I to discriminate diseases. Finally, the full RNA network was constructed with IPA database knowledge. A central node, HFN4A, has been described before as an important transcription factor of COPD-sensitive genes before in ATII cells^26^. The connection of HNF4A to other members of module I has been experimentally established by a protein-chromatin interaction study^27^. Furthermore, it was among the top five EFS-selected features. Another central node of the network was E2F1, a cell cycle-associated transcription factor (Fig. 3), which was also identified as an important feature by EFS.

### Module I can be integrated with autologous microRNA expression data

**Figure 4:**
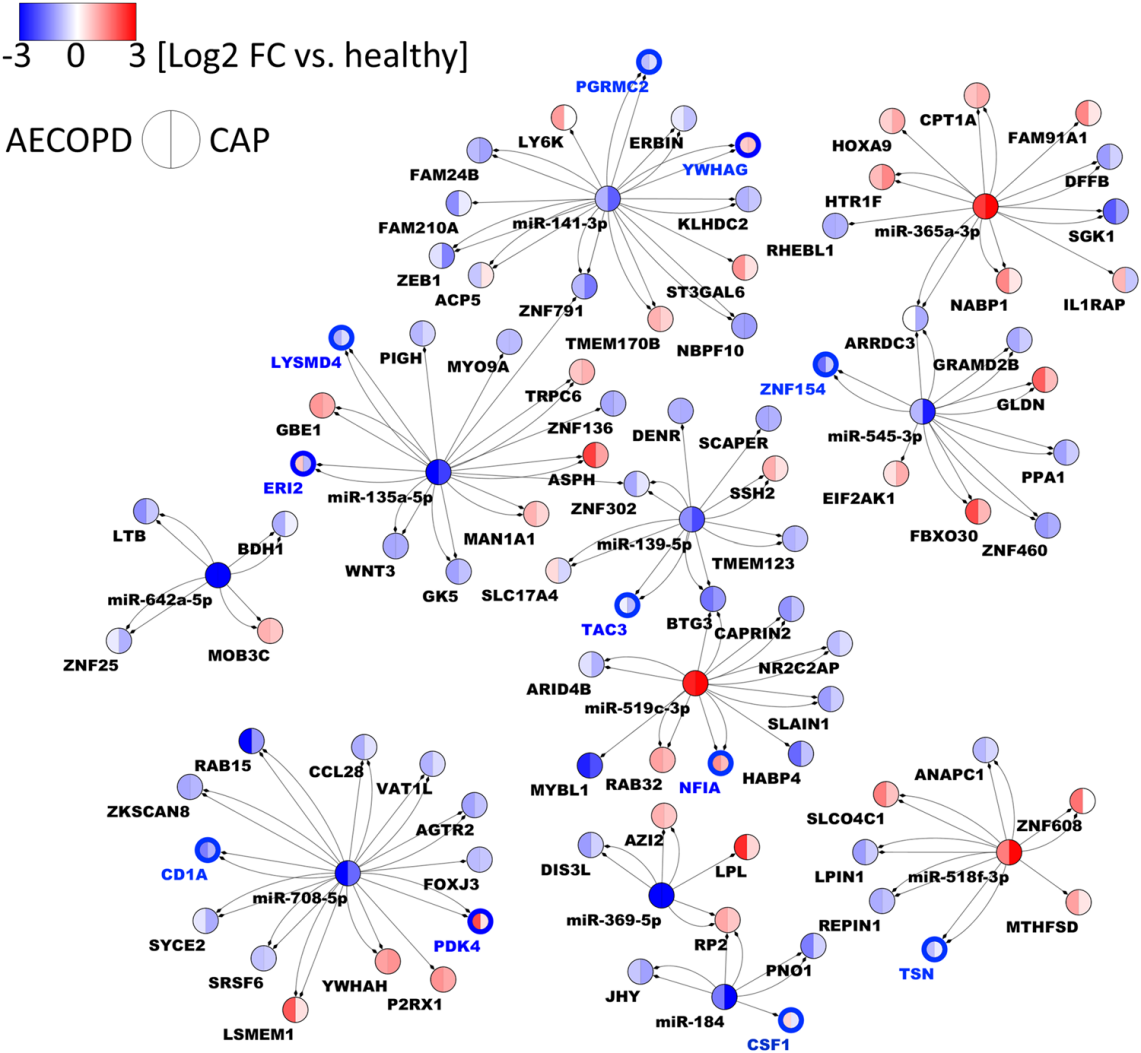
miRNA/mRNA core network of CAP/AECOPD. The entire dataset (1,621 genes) was probed for interaction with the identified miRNA pool by Ingenuity Pathway Analysis. Only experimentally validated interactions or interactions with a high prediction score (binary IPA parameter) are shown. Log_2_ fold expression changes vs. healthy control are shown for AECOPD (left semicircle) and CAP (right semicircle). Nodes circled in blue are part of module I. The network was visualized with Cytoscape v. 3.7.1.

In order to substantiate our findings and extend them to a different class of RNA, we collected the microRNA profile from all groups by Taqman Low Density Array. The microRNAs that passed visual inspection of their amplification curves and that were found to be significantly regulated between CAP and AECOPD were miR-135a-5p, miR-139-5p, miR-141-3p, miR-184, miR-365a-3p, miR-519c-3p, miR-545-3p, and miR-642a-3p. We integrated these microRNAs with the set of 1,621 genes by miRNA/mRNA target filtering into a comprehensive network of miRNA/mRNA interaction (Fig. 4). Furthermore, these miRNAs proved to be suitable discriminators of AECOPD and CAP. The five feature miRNAs as selected by EFS increased the AUC from 57.1% to 87.8% in a ROC analysis (Fig. S3).

## Discussion

Pneumonia and AECOPD are among the most common causes for acute hospitalization, and the difficulty of a timely differential diagnosis mandates the search for accessible biomarkers, as currently pneumonia tends to be underdiagnosed in COPD patients^28^. In this study, we identified RNA marker molecules in PBMCs to differentiate between healthy subjects, CAP patients and AECOPD patients, while taking PBMC composition into account. Both disease conditions showed clear differences to healthy controls as illustrated on the basis of all 1,621 coding genes that were significantly regulated in either the CAP vs. healthy or AECOPD vs. healthy contrast. Significant GO term enrichment testing of these genes yielded a strong correlation with metabolic processes and leukocyte activation. Nevertheless, the total of these differentially expressed genes was not suitable to differentiate CAP from AECOPD. The main contribution of this study is therefore the identification of a module of 120 genes (module I) that can differentiate between CAP and AECOPD.

In order to find this gene subgroup with deeper discriminative potential, we applied the WGCNA pipeline. We identified a module (termed module I) containing 120 genes, which was highly performant in discrimination of CAP and AECOPD. While many genes in this module are significantly regulated between CAP and AECOPD, as was to be expected, the cluster also contains genes that fail to pass this significance threshold. We corroborated the identified module by constructing an integrated network that highlights biologically relevant intra-modular interactions. An integral part of this network, linking the main hubs HNF4A and E2F1, is MCC, which we also identified as the most potent discriminator between CAP and AECOPD by EFS. One function of MCC is the negative regulation of canonical Wnt signalling^29^, which has been observed in the airways of COPD patients^30^. The second differentiating gene as ranked by EFS is MUC1, a member of the mucin family. MUC1 has been shown to be induced by IFNγ^31^ and IFNγ was shown to be protective in a mouse model of pneumococcal pneumonia^32^. Of note, Serum Amyloid A2 (SAA2) is also a part of this network downstream of HNF4A, and was recently described as a COPD-associated IL17 response signature gene in bronchial airway epithelial brushings^33^. We furthermore extended this network with microRNAs that were differentially expressed in CAP or AECOPD compared to healthy controls, and observed that these microRNAs have targets that are part of module I, strengthening the module’s hypothesized biological functionality. Additionally, miR-365 has been described before in the context of COPD^11^ and has been indicated as a negative regulator of pro-inflammatory IL-6 gene expression, a pro-inflammatory marker^34^. An IPA database search identified twelve targets of miR-365 that were differentially regulated between CAP and AECOPD in our dataset. Two of these, CPT1A and SGK1, are important factors in COPD pathogenesis. While CPT1A is a cigarette smoke-inducible regulator of fatty acid oxidation^35^, SGK1 increases CFTR, which might be a counter-mechanism to the observed down-regulation of CFTR in COPD^36,37^. Another miR-365 target, RHEBL1, activates mTOR signalling^38^, which controls cell proliferation via SGK1^39^. Furthermore, we found that miR-708-5p was markedly downregulated in AECOPD and CAP. This microRNA was also downregulated in the bronchial biopsies of patients with COPD after long-term treatment with inhaled corticosteroids^40^. In PBMCs, this miRNA was found to be upregulated in patients with severe vs. mild alpha1 antitrypsin deficiency (AATD), which is a genetic determinant of COPD^41^. In the same study, miR-139-5p was shown to be downregulated in AATD patients, which is a trend we also observed in AECOPD and CAP patients.

One limitation of our study is that it lacks an independent validation cohort. Such a cohort would be required to test whether the chosen approach of module finding is superior to mere significance testing. In support of our model, we can show that it increases the silhouette distance in PCA when compared to gene sets selected on the basis of significance only (Table S6) and that the identified module I is also applicable to an independent dataset. As our patient numbers are small (Table 1), the performance of the module in determining whether a patient has both CAP and AECOPD remains to be assessed.

It has been recently demonstrated that the transcriptomic differences in lung tissue in patients with COPD are largely independent from the gene expression patterns determined in whole blood^42^. Still, reproducible gene co-expression modules have been identified from whole blood of COPD patients which encourages the search for easily reachable biomarkers^43^. We have focused on AECOPD in this study, as it represents a disease manifestation with an acute therapeutical and also diagnostic need, and because it might have the potential to yield biomarkers that are absent in non-AE COPD. In CAP, while procalcitonin is an established biomarker, the need to extend the current panel has been recently stressed^44^.

Taken together, we identified a panel of protein coding transcripts in PBMCs that were differentially expressed between healthy subjects, and patients suffering from CAP or AECOPD. We identified a regulatory network of these mRNAs and regulated miRNAs, extracted central nodes of the network and retrieved a small panel of RNA markers for potential clinical application that require further validation in independent cohorts.

## Supplementary information

Appendix to Figure 1.

Figure S1.

Figure S2.

Figure S3.

Module Expression Values.

Table S1.

Table S2.
