# Supplementary material for: Transcriptional analysis identifies potential biomarkers and molecular regulators in pneumonia and COPD exacerbation
Source: Sci Rep. 2020 Jan 14;10:241. doi: 10.1038/s41598-019-57108-0 (PMC6959367; doi:10.1038/s41598-019-57108-0)
Supplement: Supplementary file 1 — Appendix to Figure 1. [file 41598_2019_57108_MOESM1_ESM.pdf]

Supplementary Information for

Transcriptional analysis identifies potential biomarkers and molecular regulators in pneumonia and COPD exacerbation

Wilhelm Bertrams, Kathrin Griss, Maria Han, Kerstin Seidel, Andreas Klemmer, Alexandra Sittka-Stark, Stefan Hippenstiel, Norbert Suttorp, Florian Finkernagel, Jochen Wilhelm, Timm Greulich, Claus F. Vogelmeier, Julio Vera, Bernd Schmeck

Correspondence: Prof. Dr. Bernd Schmeck, Institute for Lung Research, Philipps-University Marburg, Hans-Meerwein-Str. 2, 35043 Marburg, Germany, E-mail: bernd.schmeck@uni-marburg.de

<u>Appendix to Figure 1</u>

Detailed z-score heatmaps of genes from the six populated outer regions of Fig. 1C (a,b,d,f,h,i) are shown, sorted by hierarchical clustering.

a

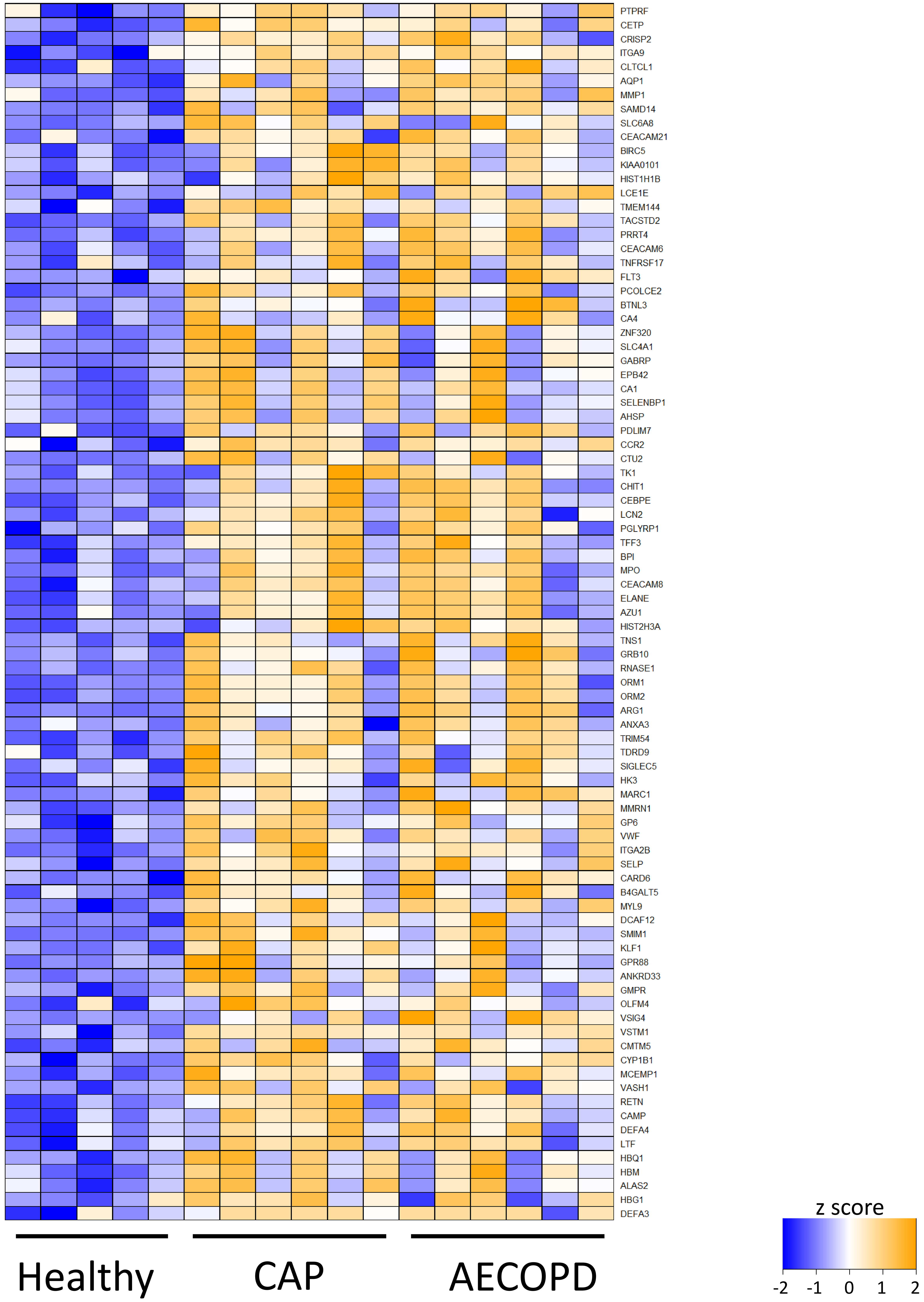

b

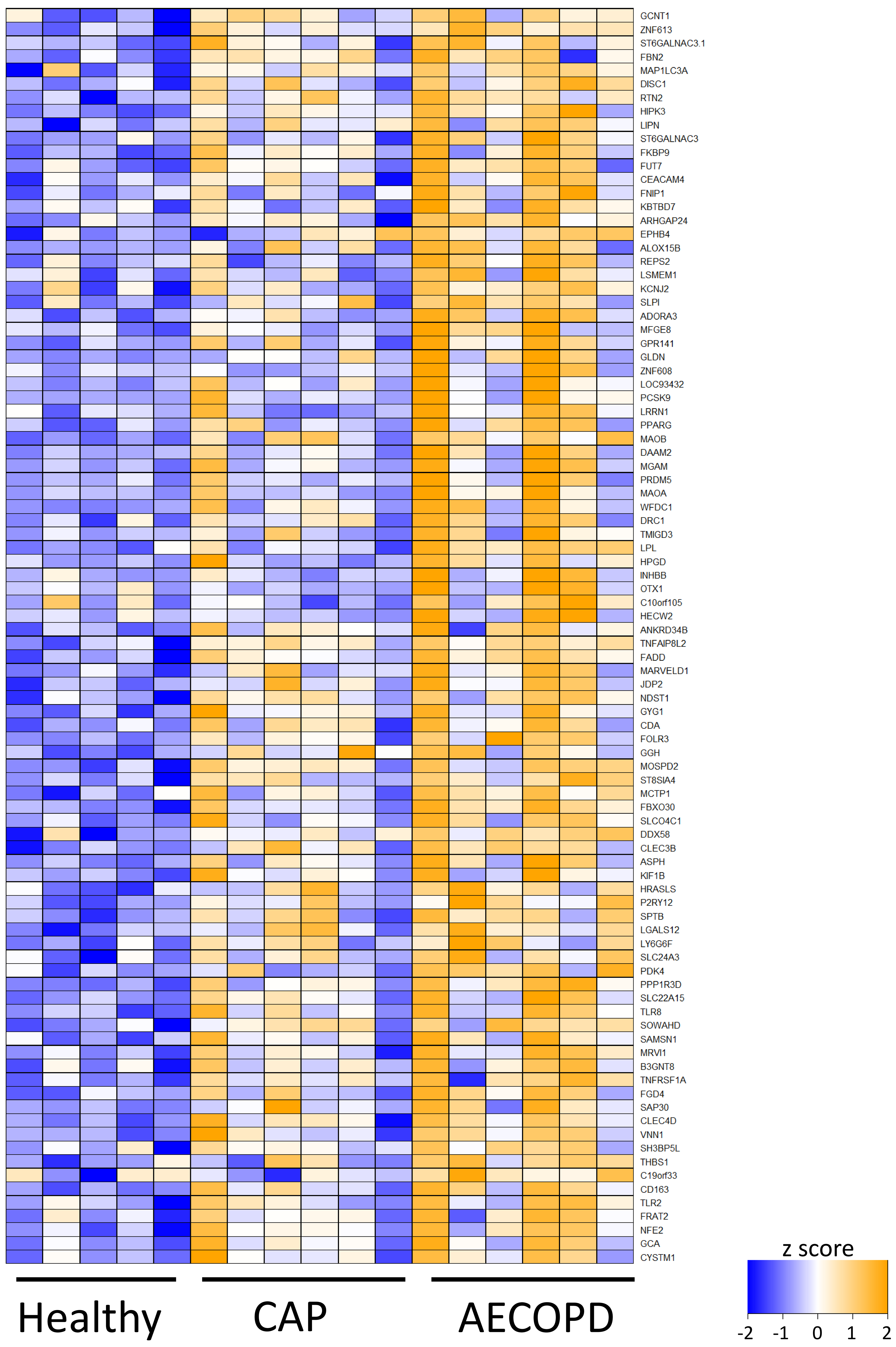
GCNT1
ZNF613
ST6GALNAC3.1
FBN2
MAP1LC3A
DISC1
RTN2
HIPK3
LIPN
ST6GALNAC3
FKBP9
FUT7
CEACAM4
FNIP1
KBTBD7
ARHGAP24
EPHB4
ALOX15B
REPS2
LSMEM1
KCNJ2
SLPI
ADORA3
MFGE8
GPR141
GLDN
ZNF608
LOC93432
PCSK9
LRRN1
PPARG
MAOB
DAAM2
MGAM
PRDM5
MAOA
WFDC1
DRC1
TMIGD3
LPL
HPGD
INHBB
OTX1
C10orf105
HECW2
ANKRD34B
TNFAIP8L2
FADD
MARVELD1
JDP2
NDST1
GYG1
CDA
FOLR3
GGH
MOSPD2
ST8SIA4
MCTP1
FBXO30
SLCO4C1
DDX58
CLEC3B
ASPH
KIF1B
HRASLS
P2RY12
SPTB
LGALS12
LY6G6F
SLC24A3
PDK4
PPP1R3D
SLC22A15
TLR8
SOWAHD
SAMSN1
MRVI1
B3GNT8
TNFRSF1A
FGD4
SAP30
CLEC4D
VNN1
SH3BP5L
THBS1
C19orf33
CD163
TLR2
FRAT2
NFE2
GCA
CYSTM1
Healthy
CAP
AECOPD
z score
-2
-1
0
1
2

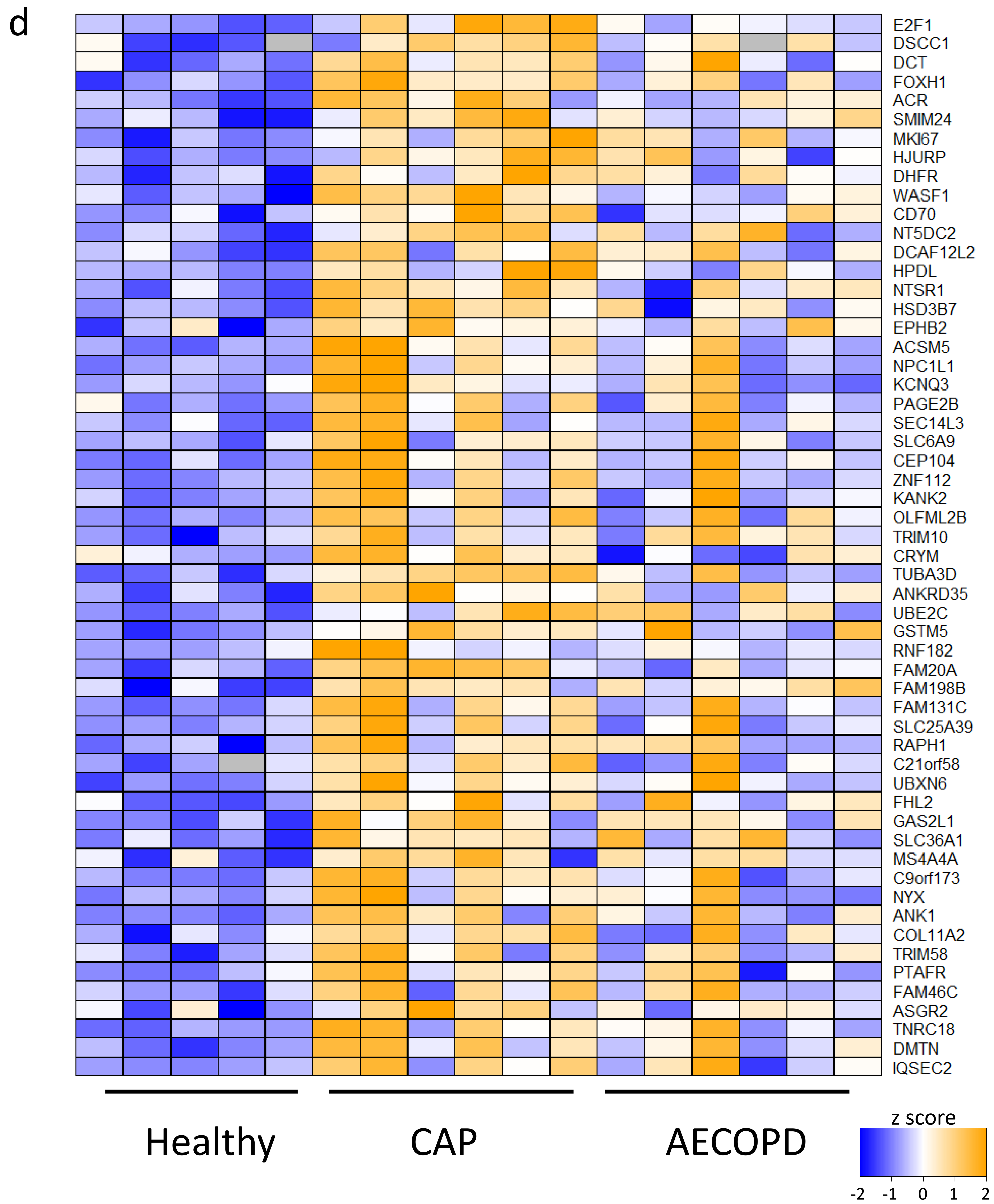
d
E2F1
DSCC1
DCT
FOXH1
ACR
SMIM24
MKI67
HJURP
DHFR
WASF1
CD70
NT5DC2
DCAF12L2
HPDL
NTSR1
HSD3B7
EPHB2
ACSM5
NPC1L1
KCNQ3
PAGE2B
SEC14L3
SLC6A9
CEP104
ZNF112
KANK2
OLFML2B
TRIM10
CRYM
TUBA3D
ANKRD35
UBE2C
GSTM5
RNF182
FAM20A
FAM198B
FAM131C
SLC25A39
RAPH1
C21orf58
UBXN6
FHL2
GAS2L1
SLC36A1
MS4A4A
C9orf173
NYX
ANK1
COL11A2
TRIM58
PTAFR
FAM46C
ASGR2
TNRC18
DMTN
IQSEC2
Healthy
CAP
AECOPD
z score
-2
-1
0
1
2

f

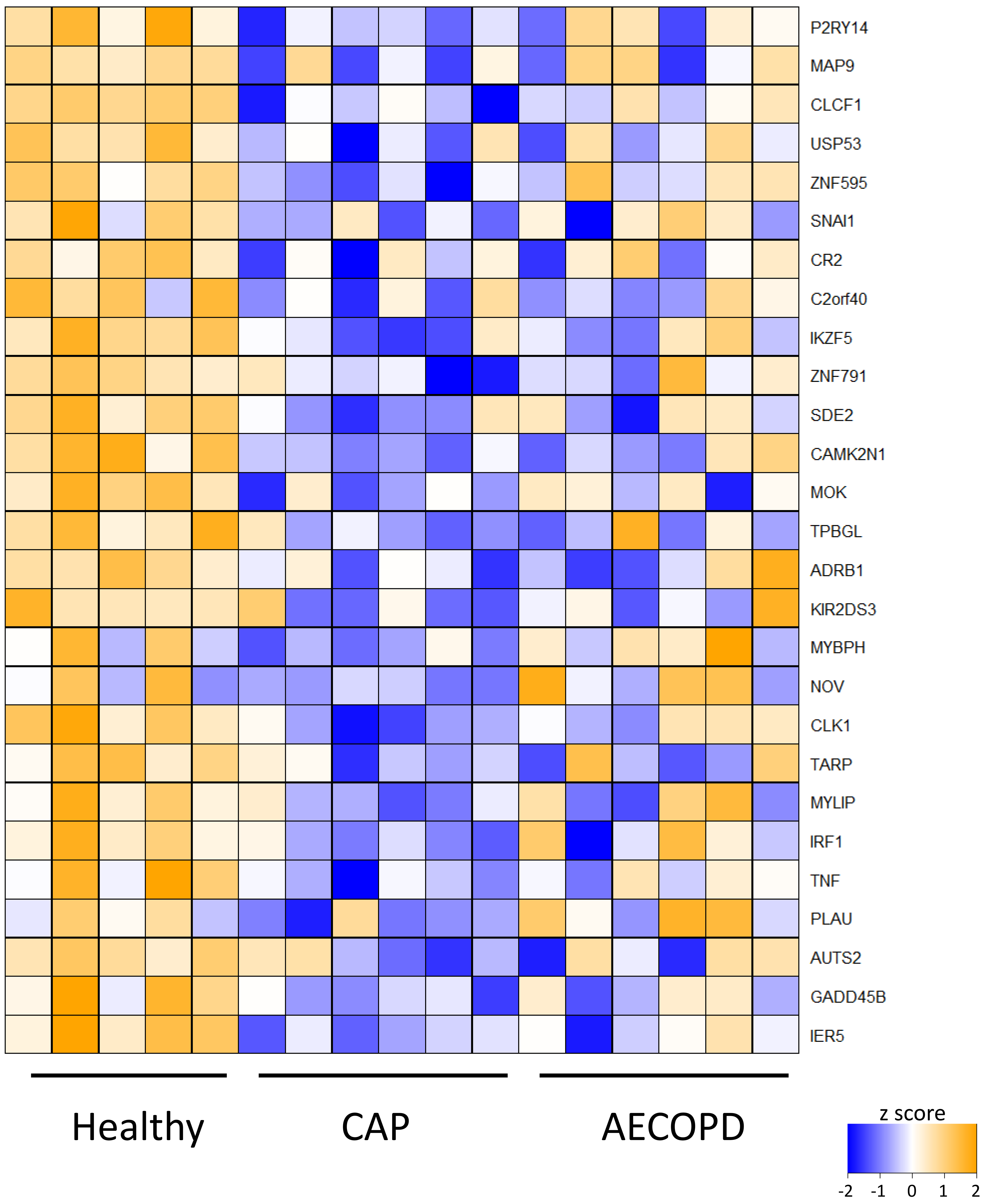
P2RY14
MAP9
CLCF1
USP53
ZNF595
SNAI1
CR2
C2orf40
IKZF5
ZNF791
SDE2
CAMK2N1
MOK
TPBGL
ADRB1
KIR2DS3
MYBPH
NOV
CLK1
TARP
MYLIP
IRF1
TNF
PLAU
AUTS2
GADD45B
IER5
Healthy
CAP
AECOPD
z score
-2
-1
0
1
2

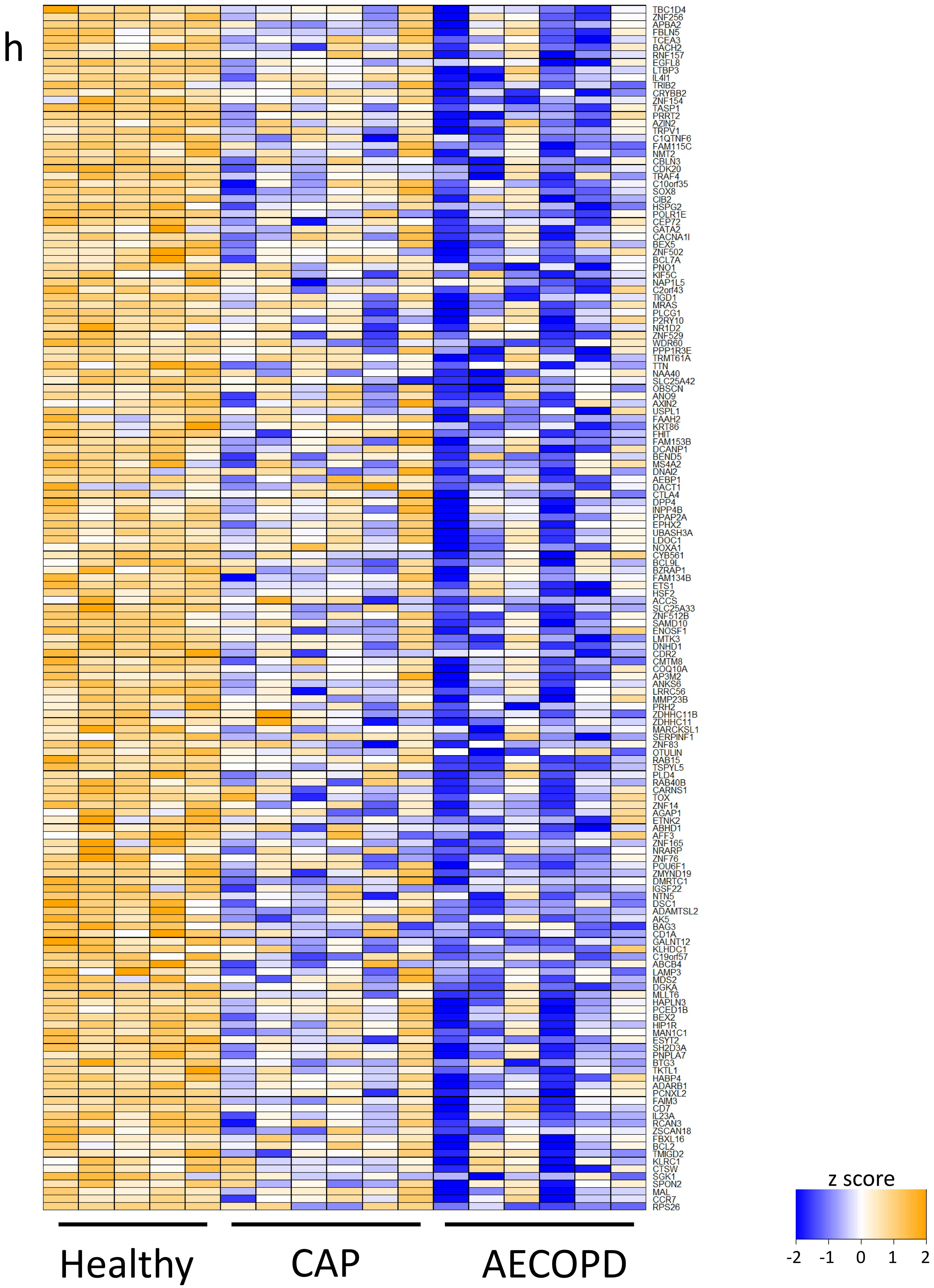
h
TBC1D4
ZNF256
APBA2
FBLN5
TCEA3
BACH2
RNF157
EGFL8
LTBP3
IL4I1
TRIB2
CRYBB2
ZNF154
TASP1
PRRT2
AZIN2
TRPV1
C1QTNF6
FAM115C
NMT2
CBLN3
CDK20
TRAF4
C10orf35
SOX8
CIB2
HSPG2
POLR1E
CEP72
GATA2
CACNA1I
BEX5
ZNF502
BCL7A
PNO1
KIF5C
NAP1L5
C2orf43
TIGD1
MRAS
PLCG1
P2RY10
NR1D2
ZNF529
WDR60
PPP1R3E
TRMT61A
TTN
NAA40
SLC25A42
OBSCN
ANO9
AXIN2
USPL1
FAAH2
KRT86
FHIT
FAM153B
DCANP1
BEND5
MS4A2
DNAI2
AEBP1
DACT1
CTLA4
DPP4
INPP4B
PPAP2A
EPHX2
UBASH3A
LDOC1
NOXA1
CYB561
BCL9L
BZRAP1
FAM134B
ETS1
HSF2
ACCS
SLC25A33
ZNF512B
SAMD10
ENOSF1
LMTK3
DNHD1
CDR2
CMTM8
COQ10A
AP3M2
ANKS6
LRRC56
MMP23B
PRH2
ZDHHC11B
ZDHHC11
MARCKSL1
SERPINF1
ZNF83
OTULIN
RAB15
TSPYL5
PLD4
RAB40B
CARNS1
TOX
ZNF14
AGAP1
ETNK2
ABHD1
AFF3
ZNF165
NRARP
ZNF76
POU6F1
ZMYND19
DMRTC1
IGSF22
NTN5
DSC1
ADAMTSL2
AK5
BAG3
CD1A
GALNT12
KLHDC1
C19orf57
ABCB4
LAMP3
MDS2
DGKA
MLLT6
HAPLN3
PCED1B
BEX2
HIP1R
MAN1C1
ESYT2
SH2D3A
PNPLA7
BTG3
TKTL1
HABP4
ADARB1
PCNXL2
FAIM3
CD7
IL23A
RCAN3
ZSCAN18
FBXL16
BCL2
TMIGD2
KLRC1
CTSW
SGK1
SPON2
MAL
CCR7
RPS26
Healthy
CAP
AECOPD
z score
-2
-1
0
1
2

i

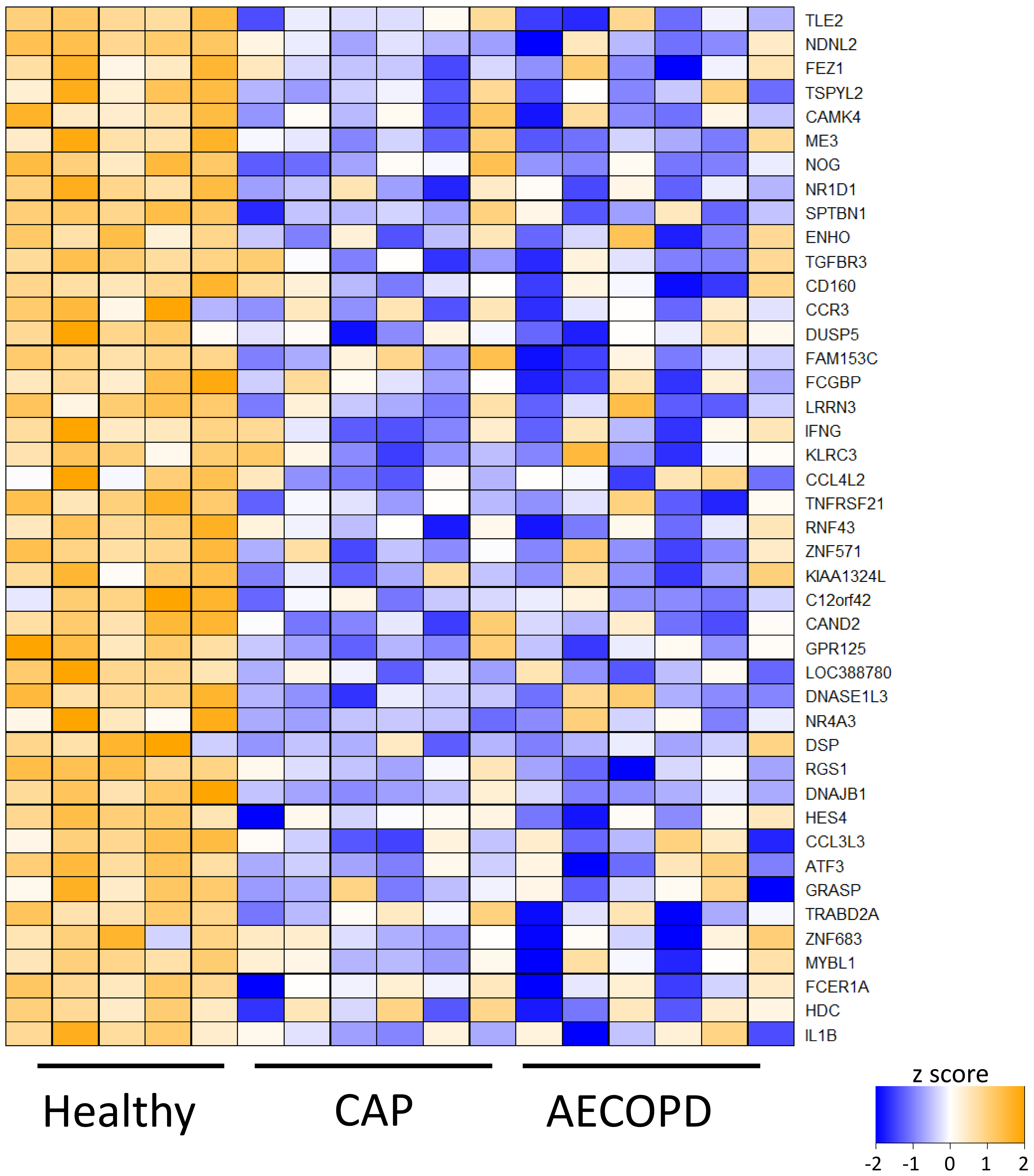
TLE2
NDNL2
FEZ1
TSPYL2
CAMK4
ME3
NOG
NR1D1
SPTBN1
ENHO
TGFBR3
CD160
CCR3
DUSP5
FAM153C
FCGBP
LRRN3
IFNG
KLRC3
CCL4L2
TNFRSF21
RNF43
ZNF571
KIAA1324L
C12orf42
CAND2
GPR125
LOC388780
DNASE1L3
NR4A3
DSP
RGS1
DNAJB1
HES4
CCL3L3
ATF3
GRASP
TRABD2A
ZNF683
MYBL1
FCER1A
HDC
IL1B
Healthy
CAP
AECOPD
z score
-2
-1
0
1
2

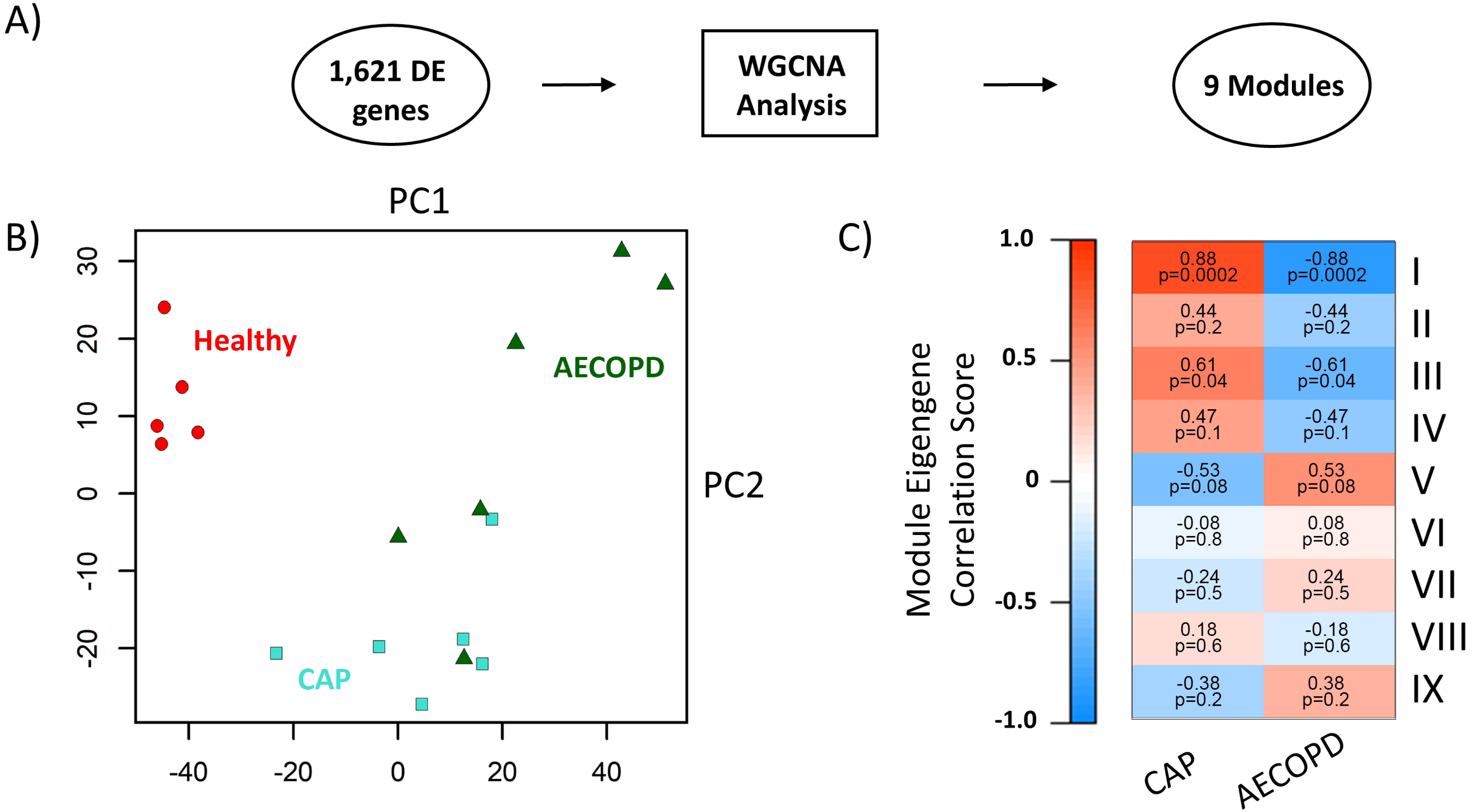

Figure S1. Stratification of all differentially expressed (DE) genes into 9 modules by co-expression analysis. Schematic representation of the workflow (A). The total of 1,621 differentially expressed genes does not clearly differentiate between CAP (turquoise squares) and AECOPD (green triangles) in PCA, while clearly set apart from healthy controls (red circles) (B). All of these genes were grouped into 9 modules of varying discriminatory potential by WGCNA. Module I showed the strongest correlationwith disease trait (correlation score -0.88, p-value 2*10-4) (C).

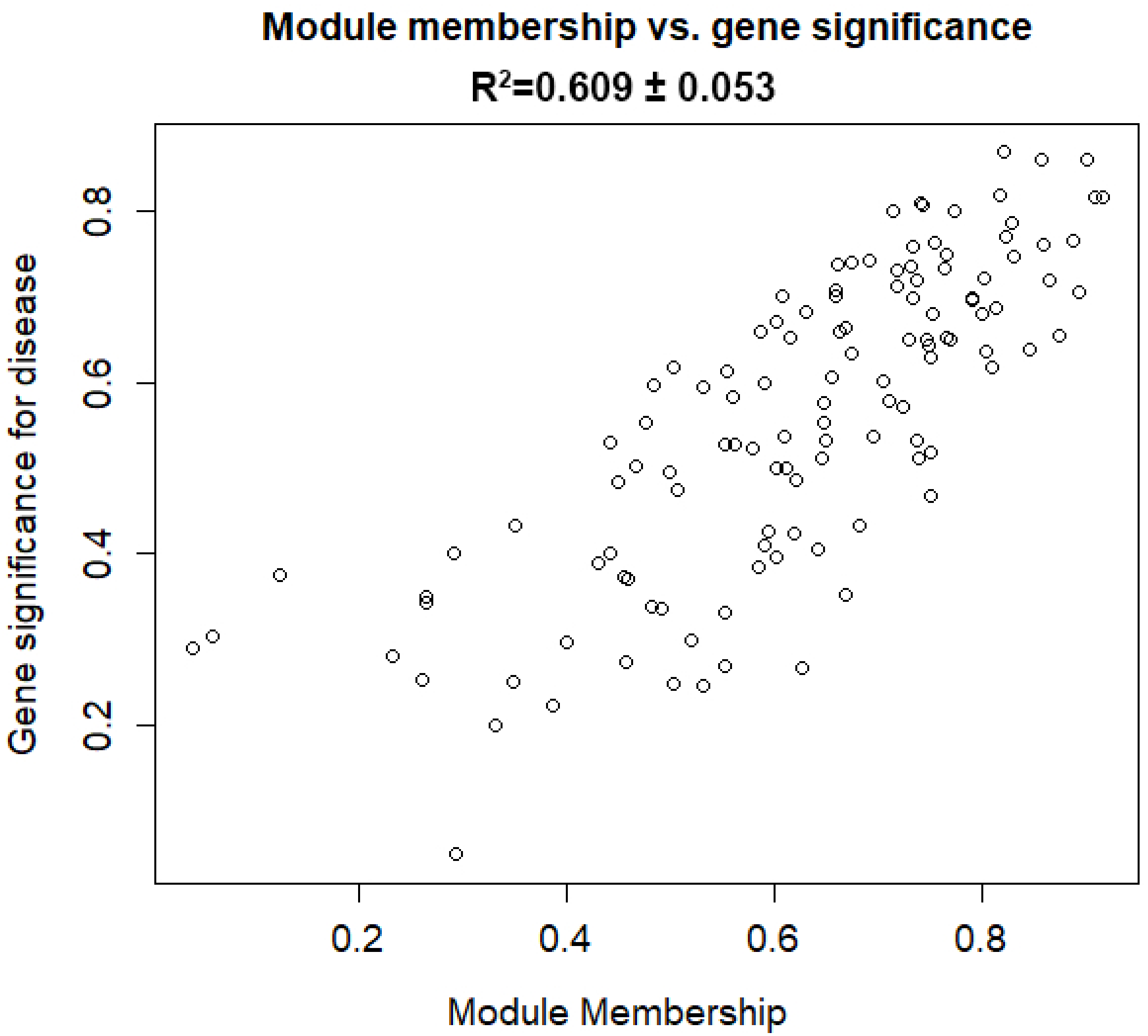

Figure S2. Intramodular analysis of gene significance and module membership in module I. Genes in module I with high module membership tend to have high significance for discrimination of CAP and COPD (disease). Gene significance for disease is computed on the basis of the Pearson correlation of expression data with the binary disease trait (CAP or AECOPD). The module membership measure is defined as the gene´s expression profile xi and the module I eigengene. Large absolute values of module membership indicate that the gene is close to (or part of) module I. The R^2 coefficient is indicated with its 95 % confidence interval.

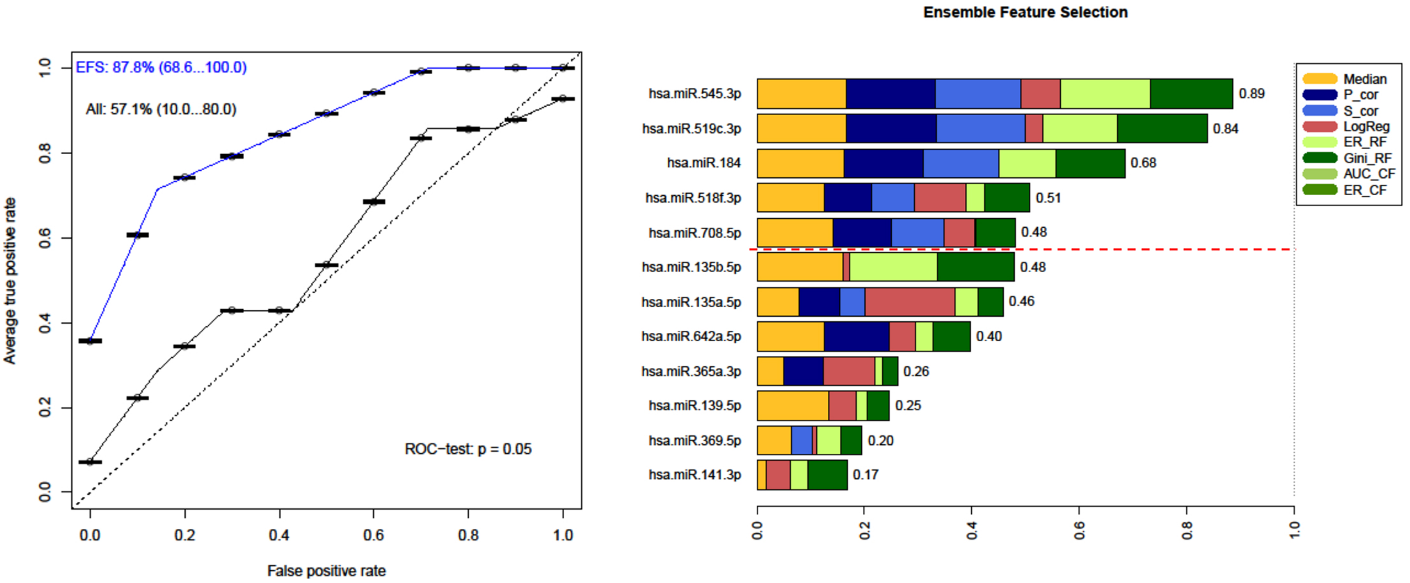

Figure S3. Selected miRNAs improve discrimination of CAP and AECOPD. While all twelve miRNAs in combination reached an area under the curve (AUC) of 57.1% in a ROC analysis, restriction to the five most important miRNAs identified by EFS above the red dashed line in (B) significantly improved the AUC to 87.8%. The average true positive rate (sensitivity, y axis) and the false positive rate (1-specificity, x axis) are shown. The black ROC curve shows the performance of all miRNAs, while the blue curve shows the performance of EFS-selected miRNAs. The dotted line indicates the performance of random guessing. Both AUC-values of the ROC curves were compared with the DeLong test as implemented in the EFS package, yielding p=0.05. ΔcT values from TLDA measurements were used for AUC computation (A). Selected features (miRNAs) are shown ranked by their importance. We used the default EFS algorithm, which builds the importance score as an additive value comprised of median comparison (p-values from Wilcoxon signed rank test), S_Cor (Spearman's rank correlation test by fast correlation filter), P_Cor (Pearson's product moment correlation test by fast correlation filter), LogReg (beta-values of logistic regression), ER_RF (Error-rate-based variable importance measure embedded in random forest) and Gini_RF (Gini-index-based variable importance measure embedded in random forest). The plot shows the relative normalized importance for each individual method (B).

Table S1. Diagnostic characteristics of the BioInflame study cohort

TLDA
CAP

BMI ± SD	25.5 ± 7.6				
Ø PSI score ± SD	82.3 ± 38.7				
PSI risk class n (%)		CURB score n (%)		CRB-65 score n (%)	
I	1 (14.3)	0	3 (42.9)	0	0 (0)
II	0 (0)	1	4 (57.1)	1	6 (85.7)
III	3 (42.9)	2	0 (0)	2	1 (14.3)
IV	2 (28.6)	3	0 (0)	3	0 (0)
V	1 (14.3)	4	0 (0)	4	0 (0)
CRP [mg/dl] ± SD	11.01 ± 6.37				

TLDA
AECOPD

BMI ± SD	23.1 ± 3.8
GOLD classification n (%)	
I	0 (0)
II	1 (14.3)
III	0 (0)
IV	6 (85.7)
n.d.	0
CRP [mg/dl] ± SD	2.05 ± 1.07

MicroArray
CAP

BMI ± SD	25.68 ± 7.60				
Ø PSI score ± SD	116.7 ± 24.6				
PSI risk class n (%)		CURB score n (%)		CRB-65 score n (%)	
I	0 (0)	0	1 (16.6)	0	0 (0)
II	0 (0)	1	5 (83.3)	1	4 (66.6)
III	1 (16.6)	2	0 (0)	2	2 (33.3)
IV	4 (66.6)	3	0 (0)	3	0 (0)
V	1 (16.6)	4	0 (0)	4	0 (0)
CRP [mg/dl] ± SD	11.57 ± 4.36				

MicroArray
AECOPD

BMI ± SD	23.07 ± 3.84
GOLD classification n (%)	
I	0 (0)
II	1 (16.6)
III	0 (0)
IV	3 (50)
n.d.	2 (33.3)
CRP [mg/dl] ± SD	2.77 ± 2.18
